# Evaluating the effect of dorsal muscle biopsies on adult Atlantic salmon growth and marine return rates

**DOI:** 10.1093/conphys/coz099

**Published:** 2020-01-17

**Authors:** Kristin Bøe, Martha J Robertson, Ian A Fleming, Michael Power

**Affiliations:** 1 Department of Ocean Sciences, Memorial University, St. John’s, NL A1K 3E6, Canada; 2 Fisheries and Oceans Canada, St. John’s, NL A1A 5J7, Canada; 3 Department of Biology, Waterloo University, Waterloo, Ontario, Canada

**Keywords:** Atlantic salmon, non-lethal biopsy, stable isotope analysis, survival, growth, feeding ecology

## Abstract

Increasing conservation and animal-welfare concerns have driven the development of non-lethal sampling of fish populations, with the use of muscle tissue biopsies now being routinely applied as a sampling method in the wild. Crucial to the success of non-lethal sampling, however, is an evaluation of the short- and long-term consequences of the treatment and ultimately the determination of how these may affect organism mortality and other fitness-related traits. The current study evaluated the use of a dorsal muscle biopsies on post-spawned Atlantic salmon emigrating to sea and undertaking a 2-month long-feeding migration before returning to spawn. Using mark-recapture, return rates and growth were compared between fish that were biopsied and externally tagged, and a control group tagged only with external tags. The biopsy treatment showed no lasting effects on fish as estimated from the two key fitness-related parameters. Results, therefore, suggest the technique can be more widely applied to gather information on marine migrating Atlantic salmon and other anadromous fishes that can be intercepted as they descend and ascend rivers during seasonal migrations. Coupled with modern tagging technologies, the use of biopsies may facilitate an improved understanding of movement and its consequences in terms of feeding patterns and growth.

## Introduction

The use of aquatic biotelemetry methods has improved our understanding of the ecology of aquatic animals by facilitating detailed monitoring of their spatial and temporal movements at multiple scales (Hussey *et al.*, 2015). With the development of biological sensor tags, aquatic biotelemetry methods can now be used to provide detailed understanding of the physiological costs of movement having direct implications for animal conservation (Wilson *et al.*, 2015) or for an improved understanding of environmental risk (Hellström *et al.*, 2016). Central to any understanding of movement ecology is an understanding of its fitness consequences, with patterns of feeding and body condition being major determinants of movement patterns (Hussey *et al.*, 2015). For example, linked telemetry and dietary trace studies have demonstrated significant covariation between the behavioural and dietary ecology of fishes at ecologically relevant timescales, with the variation amongst individuals linked to habitat carrying capacity and population niche size (Harrison *et al.*, 2017). Particularly where threatened or endangered species are concerned, the ability to gain such information in an effective non-lethal manner is critical.

The importance of understanding and quantifying the consequences of movement and habitat use for fish has resulted in the development of multiple study approaches, including the use of contaminant concentrations and dietary tracers measured in fish tissue that are now amongst the cornerstones of environmental assessment and the ecological study of fish populations (Anderson *et al.*, 2017). A key methodology for both contaminant (Jardine *et al.*, 2006) and dietary studies (Newsome *et al.*, 2007) is the application of stable isotope analysis (SIA), with fatty acid analysis (FAA) being rapidly developed as a biomarker tool (Iverson, 2009). At macro-scales, SIA can infer the spatial origin of an animal using intrinsic biological or biogeochemical markers that are particularly useful for studying animal movement because they do not require animal marking or recapture and yield time-integrated information that can be linked directly to a geographical region (Rubenstein and Hobson, 2004). FAA may in turn provide information on the spatial structure of foraging and specific or general ecological origin of energy assimilated during a feeding season (Budge *et al.*, 2006; Iverson, 2009; Bøe *et al.*, 2019). Although other non-lethal sampling methods such as the analysis of scales, mucus and fins are promising (e.g. Hutchingson and Trueman, 2006; Church *et al.*, 2009; Jardine *et al.*, 2011), white muscle tissue is most commonly used in SIA-based contaminant studies and FAA- or SIA-based dietary studies (Anderson *et al.*, 2017) because of its relevance for human consumption, long-term assimilation of contaminants (e.g. Oliveira Ribiero *et al.* 1999), and extended dietary integration period (e.g. Hesslein *et al*., 1993; Pinnegar and Polunin, 1999). Due to increasing conservation and animal-welfare concerns (e.g. Bennett *et al.*, 2016), the use of muscle tissue biopsies are routinely applied as a sampling method (e.g. Jardine *et al.*, 2011; Daly and Smale, 2013; Anderson *et al.*, 2017). Biopsies have several advantages over lethal samples, including facilitating the acquisition of a larger number of samples to generate more accurate statistical relationships (e.g. Baker *et al.*, 2004). Successive biopsies can also monitor individual levels of pollutants or dietary tracers over time, providing improved understanding of contaminant elimination rates (Walleghen *et al.*, 2013), and potentially the behavioural, environmental and physiological mechanisms important for understanding organism movement. Finally, the application of biopsies may enable regulatory authorizations to permit sampling of populations and species, where none were permitted before (Baker *et al.*, 2004).

The ultimate success of non-lethal methods, including biopsies, depends on ensuring high survival rates by minimising the injury and mortality to sampled fish. Crucial, therefore, is an evaluation of the short- and long-term behavioural and physiological consequences of the treatment and ultimately the determination of how these may affect organism mortality and other fitness-related traits. In that regard, biopsies have a long history of assessment, with the bulk of studies on teleost fishes having concluded that there is generally low (<1%) mortality (Van Meter, 1995; Evans, 2008; Schielke and Post, 2010) and few, if any, long-term sub-lethal affects on fish (Tyus *et al.*, 1999; Smith *et al.*, 2018). Most studies, however, have been conducted under laboratory conditions (e.g. Crawford *et al.*, 1977; McAndrew, 1981; Tyus *et al.*, 1999) and are of short duration, i.e. hours (Henderson *et al.*, 2016), days (Mair, 1989) or a few weeks (Crawford *et al.*, 1977; Evans, 2008). Of the studies that have addressed the longer-term lethal and sub-lethal effects of teleost fish biopsies (e.g. Leitner and Isely, 1994; Tyus *et al.,* 1999; Ackerson *et al.*, 2014), few have investigated the procedural consequences under field conditions and under ecologically-relevant time-scales, such as feeding seasons, reproductive cycles and overwintering periods. Those that have (e.g. Baker *et al.*, 2004) only indirectly assessed mortality by determining the differences in recapture probability between biopsied and non-biopsied fish (e.g. northern pike *Esox lucius*) known to be resilient to capture and handling stress (Louison *et al.*, 2017). Given the noted variability of species’ sensitivities to handling and capture, even amongst larger pelagic fishes (Mandelman and Skomal, 2009), there is a need for additional field-based assessments of the long-term consequences of biopsies, particularly for a species like Atlantic salmon, *Salmo salar*, known to exhibit environmental dependent sensitivities to handling (i.e. Kieffer *et al.*, 2002; Havn *et al.*, 2015).

The current study investigates the growth and return of post-spawned wild Atlantic salmon subject to a biopsy treatment prior to the initiation of the marine feeding season. Atlantic salmon is an anadromous salmonid subject to conservation concerns as many populations now are imperilled, largely due to reduced survival at sea (Parrish *et al.*, 1998; Chaput, 2012; Mills *et al.*, 2013). Knowledge gaps surrounding Atlantic salmon marine ecology and mortality sources have resulted in considerable research effort, including numerous studies that have applied SIA sampling (e.g. Dempson *et al.*, 2010; Kelly *et al.*, 2018) or telemetry methods (e.g. Hedger *et al.*, 2017; Strøm *et al.*, 2017; Lothian *et al.*, 2018) for improved understanding of marine feeding and movement ecology. Combining methods, knowing that biopsies held minimal consequences for long-term survival and growth would, therefore, enhance overall abilities to study wild Atlantic salmon. Therefore, the aims of this study were to determine: (i) if the migratory return rate was lower in biopsied compared to non-biopsied Atlantic salmon kelts, and (ii) if the growth of biopsied Atlantic salmon kelts that returned to spawn was lower compared to non-biopsied fish.

## Methods

The study was conducted in a North American Atlantic salmon population from the Campbellton River, a medium sized river (drainage area: 296 km^2^) located on the northeast coast of Newfoundland (Canada, 49.2°N, 54.9°W). The river is dominated by small salmon (<63 cm fork length, *L_F_*) maturing after one winter at sea (1SW) (89–94% of total salmon returns), typically at 50–55 cm *L_F_* (Downton and Reddin, 2004). Salmon 63 cm *L_F_* or larger are almost exclusively consecutive repeat spawners, which overwinter in freshwater as kelts and emigrate to sea in spring for an average feeding period of 2 months before reconditioned individuals return to repeat spawn (Downton and Reddin, 2004). The migration pattern of this life history contrasts to that of maiden 1SW and alternate repeat spawners, which spend on average 14 months at sea (Klemetsen *et al*., 2003). A Department of Fisheries and Oceans (DFO) operated counting facility records on average 1500 (±673 SD) downstream migrating post-spawned adults (kelts) annually (2002–2014), of which ~300 are tagged with external Floy T-bar anchor tags as part of a population monitoring programme. This work has demonstrated that annual return rates of consecutive spawners to Campbellton River are variable, ranging from 9 to 40% (Downton and Reddin, 2004).

**Figure 1:**
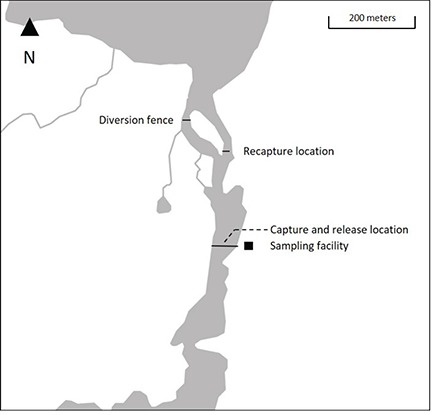
Map of the Campbellton River study site with location of capture and release, sampling and recapture of Atlantic salmon kelts.

Collection of Atlantic salmon tissue was obtained from kelts as they descended Campbellton River in spring 2016 during the seaward migration. Downstream migrating individuals were intercepted at the DFO operated fish counting facility located ~500 m from the river mouth (Fig. 1). Kelts that underwent the biopsy treatment were moved from the trap to a sampling facility located ~5 m from the counting facility and anaesthetized in an induction bath of clove oil. A blue external T-bar tag with information on fish identification and return reward in the event it was intercepted by the fishery was fitted to each fish before fork length (*L_F_*) was measured. Three to five scales were then removed to expose a ~0.5 x 0.5 cm area of skin anterior to the dorsal fin and above the lateral line before a 4 mm disposable biopsy punch (Milltex®) was inserted, removing ~50 mg of dorsal muscle. Wounds were not disinfected as the use of topical antiseptics may disrupt the cutaneous mucus layer of the fish allowing easier penetration by pathogens (Wagner and Cooke, 2005). Following the biopsy procedure, post-sampling monitoring was completed for 30 minutes–1 hour to ensure complete recovery before individuals were released back into the river.

**Figure 2:**
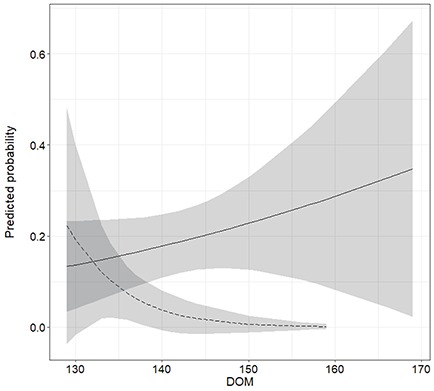
Predicted probability of an Atlantic salmon kelt returning as a consecutive spawner to Campbellton River in the summer of 2016 plotted as a function of DOM and MH (solid line: consecutive spawners, dashed line: first time spawners). Shaded area represents the 95% prediction confidence intervals.

The control group consisted of downstream migrating fish intercepted at the counting facility, measured for fork length and fitted with a yellow external T-bar tag carrying fish identification and reward information. Being part of a DFO population assessment programme, these fishes were measured and tagged within the trap of the counting facility without anaesthesia to comply with the sampling protocol used in Campbellton River monitoring programme. Tag identification and length were recorded and measured from kelts tagged in the monitoring programme in previous years and intercepted at the counting facility during the 2016 downstream migration and were also included in the control group.

To account for potential carry-over effects from previous migration history (MH; i.e. maiden vs. repeat spawners) on kelt condition and return probabilities, only fish with known migration histories based on scale pattern analysis or mark-recapture history from the downstream migration in spring 2015 were included in analyses. Due to a low sample size of alternate repeat spawners, reflecting their overall low abundance in Campbellton River (Downton and Reddin, 2004), these were excluded from analysis. Potential biases due to the presence of kelts tagged prior to 2016 in the control group were investigated by comparing the growth and return rates to the control fish tagged in 2016.

Marine growth and return rates of the treatment and control groups were assessed by the size and number of externally tagged fish intercepted at the counting facility during the upstream migration in summer 2016. Because the recreational fishery in Campbellton River only occurs upstream of the counting facility (Downton, 2001), the proportion of fish intercepted during the upstream migration was considered an accurate representation of the marine return rate. Although this rate cannot directly be interpreted as survival because it is confounded by repeat spawning strategy (consecutive vs. alternate spawning), the numerical dominance of consecutive compared to alternate spawners in the population suggests that the rate can be considered an approximation of it.

All upstream migrating salmon entering the trap were examined for an external tag via a video monitoring system (Panasonic, Colour CCTV Camera WV-CP294, USA, Newark) installed to count all returning Atlantic salmon. When a tagged fish was identified, the fish was removed and measured for length (cm), mass (kg) and tag identification information was collected. Scales were also collected for the determination of spawning history and repeat spawning strategy following standardised international guidelines for Atlantic salmon (ICES, 1992).

### Analyses

Data inspection and statistical analyses were performed in the statistical software R (version 3.4.4, www.r-project.org). In all analyses, model suitability was assessed by visual inspections of residuals for indications of violations of model assumptions, e.g. normality, variance homogeneity (Sokal and Rohlf, 1973).

The return rate of kelts to consecutive repeat spawning was calculated as the proportion of tagged fish in the control and treatment groups leaving the system in spring 2016 that returned during the upstream migration the same summer. The effect of the biopsy treatment on the return rate was assessed by testing the statistical significance of the variable in a generalised linear model that treated the return status of individual fish tagged during the 2016 downstream migration as a binomial response variable (e.g. returned, did not return) using a logit link function. As previous work has suggested an association between Atlantic salmon size and repeat spawning rates (Jonsson *et al.*, 1991, 1997), we also investigated the potential effects of fish length and possible interactions with treatment on the return probability of kelts by including the continuous variable fork length (*L_F_*, cm) at release. Migration timing may also be correlated to migration success in Atlantic salmon (Birnie-Gauvin *et al.*, 2019), and to account for potential migration timing effects we included the day of migration (DOM) as a covariate. To investigate possible effects of prior spawning history on return probabilities of kelts, the factorial term for MH distinguishing between first-time and consecutively spawned kelts was included. To reduce model complexity and collinearity arising from the correlation of kelt length and spawning history, the two terms were tested separately. The full statistical models considered were:

$$ {{{Y}}}_{{{i}{jk}}}&\!\!\!=\!\!\!&{\mathrm{Treatment}}_{{{i}}}+{{{L}}}_{{{Fi}}}+{\mathrm{DOM}}_{{{i}}}+{\mathrm{Treatment}}_{{{i}{jk}}} \times\\ && {{{L}}}_{{{Fi}}}+{\mathrm{Treatment}}_{{{i}{jk}}} \times{\mathrm{DOM}}_{{{i}}}+{{{L}}}_{{{Fi}}} \times{\mathrm{DOM}}_{{{i}}} $$

and

$$ {{{Y}}}_{{{i}{jk}}}&\!\!\!=\!\!\!&{\mathrm{Treatment}}_{{{i}}}+{\mathrm{MH}}_{{{i}{jk}}}+{\mathrm{DOM}}_{{{i}}}+{\mathrm{Treatment}}_{{{i}{jk}}} \times\\&& {\mathrm{MH}}_{{{i}{jk}}\ {{i}}}+{\mathrm{Treatment}}_{{{i}{jk}}} \times{\mathrm{DOM}}_{{{i}}}+{\mathrm{MH}}_{{{i}{jk}}} \times{\mathrm{DOM}}_{{{i}}}, $$

where *Y_ijk_* is the return status of individual fish *i* exposed to treatment *j* (control, biopsy) of spawning history *k* (first-time spawned, consecutively spawned)*, L_Fi_* is the fork length of fish *i* at release and DOM*_i_* is the DOM of fish *i* (the day that an individual kelt was intercepted at the counting facility). A step by step approach of model simplification from the full model using backward elimination was followed, with the Akaike information criterion (AIC) and Akaike weights (*w_i_*) used to select the “best” model (Burnham *et al.*, 2002; Anderson, 2008) and the significance of estimators tested with likelihood ratio tests (LRT) (Sokal and Rohlf, 1995). The *w_i_* term can be interpreted as the probability that model *i* is the best approximating model for the data within the candidate set of models considered (Anderson, 2008).

**Table 1:** Summary information regarding numbers of sampled Atlantic salmon kelts in the treatment and control groups (numbers of returning fish in brackets), for k length (*L_F_*), Julian DOM and marine residency time (days)

Treatment	First time spawners	Consecutive spawners	Total	*L_F_*		*L_F_*	DOM		DOM	Marine residency (mean + SD)	Residency range
				(mean ± SD)		range	(mean ± SD)		range		
Biopsy	47 (3)	31 (7)	78 (10)	58.7 ± SD 6.1		48.0–73.0	138.1 ± 2.5		133–141	69.7 ± 21.0	50–107
Control	80 (3)	76 (17)	156 (20)	57.4 ± SD 6.6		40.6–78.8	143.1 ± 9.0		129–169	62.7 ± 18.4	41–102

**Table 2:** AIC model selection table containing the ten best ranked binomial models fit to explain Atlantic salmon kelt return probability to Campbellton River. MH = migration history, DOM = Julian day of migration and treatment = biopsy or control

Model	df	logLik	AIC	dAIC	Weight
*y* ~ MH + DOM + MH x DOM	4	−81.5	171.0	0.0	0.370
*y* ~ Treatment + MH + DOM + MH x DOM	5	−81.5	172.9	−2.0	0.139
*y* ~ MH	2	−84.7	173.4	−2.4	0.114
*y* ~ MH + DOM + Treatment + DOM x Treatment + MH x DOM	6	−81.0	174.1	−3.1	0.079
*y* ~ MH + DOM + Treatment + MH x DOM + MH x Treatment	6	−81.1	174.2	−3.2	0.073
*y* ~ MH + Treatment	3	−84.6	175.3	−4.3	0.043
*y* ~ MH + Treatment + MH x Treatment	4	−83.7	175.3	−4.3	0.043
*y* ~ MH + DOM	3	−84.7	175.3	−4.3	0.042
*y* ~ MH + DOM + Treatment + MH x Treatment + DOM x Treatment	7	−81.0	175.9	−4.9	0.031
*y* ~ MH + DOM x Treatment	5	−83.6	177.3	−6.3	0.016

Growth was assessed as the difference between final (re-capture) and initial (release) length (*L_f_*, cm) and compared between the biopsied and control groups using a general linear model. Because growth in fish depends in part on the size of the fish (Eberhardt and Ricker, 1977), and may be influenced by time spent at sea (Jonsson and Jonsson, 2014), fish length and at sea residency were included as covariates. The interactions considered were treatment x log (FL), treatment x residency and log (FL) x residency and the full model was as follows:

$$ \mathrm{Log}\ \left({{{y}}}_{{{i}{jk}}}\right)&\!\!\!=\!\!\!&{\mathrm{Treatment}}_{{{i}{jk}}}+\mathrm{\log}\ \left({{{L}}}_{\mathrm{Fi}}\right)+{\mathrm{Residency}}_{{{i}}}+\\&&{\mathrm{Treatment}}_{{{i}{jk}}}\times\ \mathrm{\log}\ \left({{{L}}}_{\mathrm{Fi}}\right)+{\mathrm{Treatment}}_{{{i}{jk}}}\times\\&& {\mathrm{Residency}}_{{{i}}}+\mathrm{\log}\ \left({{{L}}}_{\mathrm{Fi}}\right)\times{\mathrm{Residency}}_{{{i}}}+{{{e}}}_{{{i}}}, $$

where *y_ijk_* is the growth in centimetre of individual fish *i* treatment*_j_*, *L*_Fi_ is as defined above and Residency*_i_* is the number of days between the capture and release on the downstream migration and the recapture during the upstream migration. *e_i_* represents the normally distributed error term. As the ratio *n*/*k* did not exceed 40 (Burnham *et al.*, 2002), model simplication from the full model was followed using the AIC for small sample sizes (AICc) and Akaike weights, with the significance of estimators tested with *F*-tests.

## Results

In total, 78 biopsied (mean ± SD *L_F_*: 58.7 ± 6.1 cm, range: 48.0–73.0 cm) and 156 control kelts (*L_F_*: 57.4 ± 6.6 cm, range: 40.6–78.8 cm) with known MH were intercepted between 8 May and 17 June during the downstream migration in spring 2016. Scale pattern analysis and mark-recapture history determined that 51 and 60% of the individuals consisted of first-timed spawned kelts in the treatment and control groups, respectively, the rest being consecutive repeat spawners (Table 1). No significant difference in size was found between the two groups (*F*_1,232_ = 2.1, *P* = 0.15). No mortalities were recorded in the treatment or control group during downstream tagging and all fishes were deemed sufficiently recovered prior to release.

### Kelt returns

In total, 30 externally tagged kelts from the treatment (*n* = 10, 12.8%) and control (*n* = 20, 12.8%) groups returned to the DFO counting facility between 5 July and 30 August 2016.

Amongst the models fit to return status of biopsied and control kelts, the AIC criterion provided most support for an interaction between MH and DOM (Table 2), predicting a positive effect of DOM on return probability in consecutive spawners and a negative effect in first-time spawners (Fig. 2). Although the AIC criterion did not distinguish between this model and a model also containing the treatment term, the Akaike information weights (*w_i_*) indicated there was a <14% chance of the model including treatment being correct (Table 2). Furthermore, the treatment effect as estimated was not significant (Table 3).

**Figure 3:**
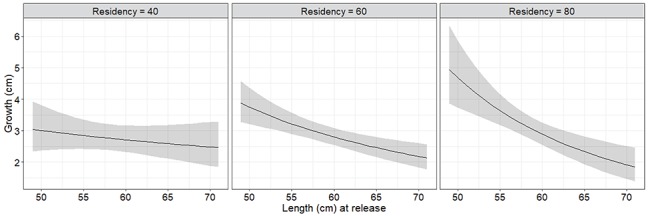
Predicted growth (length, cm) of Atlantic salmon kelts that returned to Campbellton River as consecutive spawners in the summer of 2016 plotted as a function of fork length at release and marine residency (days). Shaded area represents the 95% prediction confidence intervals.

A comparison of return rates between control kelts tagged prior to, or during, spring 2016 revealed a significantly higher return probability in the former group (binomial model, *χ*^2^ = 11.56, *P* < 0.001). When the analysis was limited to consecutive spawners, no difference was found (binomial model, *χ*^2^ = 2.01, *P* = 0.16). The lack of difference in return rates in consecutive spawners suggested that the significant difference in the full dataset was due to the fact that fish tagged prior to 2016 consisted exclusively of consecutive spawners with a higher proportion of first-time spawners amongst those tagged in 2016.

**Table 3:** Parameter estimates (logit scale) and LRT statistics for the two most supported models fitted for explaining return probabilities of tagged Atlantic salmon kelts to Campbellton River. MH = migration history, DOM = Julian day of migration and treatment = biopsy or control

	Parameter estimates			Likelihood ratio test statistics			
Model	Term	Coefficient	SE	Effect	df	*X* ^2^	*P*
*y* ~ MH + DOM + MH x DOM	Intercept	−5.89	3.84	DOM	1	0.08	0.77
	DOM	0.03	0.03	MH	1	9.81	0.00
	MH	28.03	13.84	DOM x MH	1	6.34	0.01
	DOM x MH	−0.21	0.10				
*y* ~ Treatment + MH + DOM + MH x DOM	Intercept	−5.72	3.90	Treatment	1	0.00	1.00
	Treatment	0.09	0.44	DOM	1	0.09	0.76
	DOM	0.03	0.03	MH	1	9.85	0.00
	MH	27.75	13.75	DOM x MH	1	6.33	0.01
	DOM x MH	−0.21	0.10				

**Table 4:** AICc model selection table containing the 10 best ranked models fit to explain Campbellton River Atlantic salmon kelt growth (cm) during the marine migration period, where treatment = biopsy or control and residency defines length or the marine residency period

Model	df	logLik	AICc	dAICc	Weight
log (*y*) ~ log (*L_F_*) + Residency + log (*L_F_*) x Residency	5	1.1	10.4	0.0	0.317
log (*y*) ~ log (*L_F_*)	3	−2.2	11.3	1.0	0.196
log (*y*) ~ log (*L_F_*) + Residency	4	−1.3	12.1	1.7	0.134
log (*y*) ~ Treatment + log (*L_F_*) + Residency + log (*L_F_*) x Residency	6	1.7	12.2	1.8	0.128
log (*y*) ~ log (*L_F_*) + Treatment	4	−1.7	13.0	2.6	0.085
log (*y*) ~ log (*L_F_*) + Residency + Treatment + log (*L_F_*) x Residency + Residency x Treatment	7	2.1	14.9	4.5	0.033
log (*y*) ~ log (*L_F_*) + Treatment + log (*L_F_*) + log (*L_F_*) x Treatment	5	−1.4	15.4	5.0	0.026
log (*y*) ~ log (*L_F_*) + Residency + Treatment + log (*L_F_*) x Residency + log (*L_F_*) x Treatment	7	1.8	15.6	5.2	0.024
log (*y*) ~ Residency + log (*L_F_*) + Treatment + log (*L_F_*) x Treatment	6	−0.1	15.9	5.5	0.020
log (*y*) ~ log (*L_F_*) + Residency + Treatment + Residency x Treatment	6	−0.3	16.3	5.9	0.017

**Table 5:** Parameter estimates and *F* test statistics for the two most supported models fitted to marine growth (cm) of Atlantic salmon kelts that returned to Campbellton River, where treatment = biopsy or control and residency defines length or the marine residency period

	Parameter estimates			*F*-test			
Model	Term	Coefficient	SE	Effect	df	*F*	*P*
log (*y*) ~ log (*L_F_*) + residency + log (*L_F_*) x residency	Intercept	−5.32	6.25	log (*L_F_*)	1	14.59	0.00
	log (L_F_)	1.53	1.54	Residency	1	2.00	0.17
	Residency	0.22	0.10	log (*L_F_*) x Residency	1	4.33	0.05
	log (*L_F_*) x Residency	−0.05	0.03				
log(*y*) ~ log (*L_F_*)	Intercept	7.47	1.79	log (*L_F_*)	1	12.64	0.00
	log (*L_F_*)	−1.57	0.44				
log(*y*) ~ log (*L_F_*) + Residency	Intercept	7.21	1.78	log (*L_F_*)	1	12.99	0.00
	log (*L_F_*)	−1.56	0.44	residency	1	1.78	0.19
	Residency	3.43e^−3^	2.57e^−3^				
log (*y*) ~ Treatment + log (*L_F_*) + residency + log (*L_F_*) x residency	Intercept (biopsy)	−5.18	6.24	Treatment (control)	1	4.41	0.05
	Treatment (control)	0.11	0.10	log (*L_F_*)	1	11.32	0.00
	Log (*L_F_*)	1.47	1.54	Residency	1	2.68	0.11
	Residency	0.20	0.10	log (*L_F_*) x Residency	1	3.76	0.06
	log (*L_F_*) x Residency	−0.05	0.03				

### Kelt growth upon return as repeat spawners

Kelts grew on average 3.13 cm (±0.94 SD) during the period of marine residency which on average lasted 65 days (±19.2 SD), and this corresponded to a 5.53% increase in length (±2.13 SD). The AICc criterion for log-transformed growth did not distinguish between the four best ranked models, of which one included the treatment term (Table 4). The treatment term was, however, not statistically significant and there was a 13% or less chance the model was correct based on *w_i_*, compared to the highest ranked model with a *w_i_* of 32% (Tables 4 and 5). The best supported AICc model for log-transformed growth depended on the interaction between fish fork length at release and the duration of the marine residency (Tables 4 and 5), with steeper declines in growth observed as a function of larger size as the duration of the sea residency increased (Fig. 3). Marine residency did not differ between biopsied and control kelts (*F*_1,28_ = 0.86, *P* = 0.36). No difference in growth was found between kelts tagged prior to, or during, spring 2016 (*F*_1,18_ = 1.07, *P* = 0.31).

## Discussion

Data obtained from migrating kelts in the Campbellton River showed no effect of biopsies on marine return rates as measured by the number of individuals tagged and re-captured at a counting fence operated in the lower reaches of the river. Return rate, however, did appear to depend on the interaction between MH and DOM, with their being higher return probabilities for consecutive spawners than first-time spawners, except during the early portion of the migration period. Although the return rates measured in the current study are a product of both survival and repeat spawning strategy, the low numbers of alternate repeat spawners found in Campbellton River in general suggests that return rates to annual spawning closely approximate survival. Although growth did depend on the length of the fish and the time it spent in the marine environment, no growth-related effects were observed as a function of the biopsy treatment. Combined, the results indicate that biopsies lead to no measurable long-term effects on key fitness parameters such as survival and growth and that use of biopsies in studies will not bias data in any meaningful way.

Consistent with shorter-term survival studies on a wide variety of species, survivorship (as measured by return rates) associated with biopsies were high, with values of >99% not being uncommon for other teleost species (Schielke and Post, 2010) even when fishes were subjected to multiple biopsy events (Tyus *et al.*, 1999). Whilst the data suggest the acute responses of biopsied fish are minimal, rates do vary amongst species and may be affected by environmental and other factors. For example, Mair (1989) noted that the technique may be size-limited, being safe only for fish of a standard length >120 mm. Size clearly favours biopsy use, with the practise being especially pronounced in elasmobranch studies (e.g. Heupel and Simpfendorfer, 2010; Hammerschlag and Sulikowski, 2011), where long-term effects are known to be minimal and short-term injury or haemorrhaging issues have largely been addressed (Meyer *et al.*, 2018). Warmer waters may also pose issues for biopsy use, with reported mortalities for tested coral reef species (*Plectropomus maculatus* and *Lutjanus carponotatus)* as high as 3% (Evans, 2008), possibly as a result of the higher likelihood of infection when biopsy procedures are used in more pathogen-rich environments (Jardine *et al.*, 2011). The degree of invasiveness also appears to correlate with mortality, with muscle only biopsies having much lower mortality than combined muscle and liver biopsies, where survival was reduced to 90% after 11 weeks (Leitner and Isely, 1994). With suitable care and handling of fish during the biopsy procedure, results reported here and elsewhere in the literature indicate the use of biopsies have minimal to no mortality implications for larger, cold water fish.

Growth has similarly been assessed for multiple species with results generally pointing to no, or minimal, impact. Six week follow-up studies of biopsied smallmouth bass (*Micropterus dolomieu*) showed slightly negative but statistically non-significant effects on growth (Ackerson *et al.*, 2014), whereas long-term studies with rainbow trout (*Oncorhynchus mykiss*), razorback sucker (*Xyrauchen texanus*) and bonytail chub (*Gila elegans*) showed no differences in condition or growth between biopsied and non-biopsied fish held in a hatchery facility (Tyus *et al.*, 1999). The number of studies reporting a lack of short- and long-term responses in growth is encouraging as growth is typically more responsive to long-term chronic stresses and, as a consequence, has typically been used in assessing the chronic effects of stress regimes on fish (e.g. Adams 2002). Those studies having investigated long-term growth effects of biopsied fish, however, have done so on fish held in captivity (Tyus *et al.*, 1999), and as a consequence, have not accounted for potential synergistic effects of natural stressors such as predation, harvesting and food availability. Thus, the first reported absence of a long-term effect for growth in the wild, as found here, provides stronger evidence for the minimal effects of biopsies in field studies.

Although previous work on short-term influences of biopsy treatment hints at differences in responses amongst species (e.g. Tyus *et al.*, 1999; Henderson *et al.*, 2016), there is no direct account of this in the literature. As such, there is a need for more research on the phylogenetic factors that may contribute to variability in responses amongst species non-lethally biopsied for muscle or other tissue and on how environmental factors such as temperature may affect healing and the likelihood of infection. Before combining telemetry and biopsy methods on biopsy-tolerant species, direct testing of possible interaction effects should also be performed, particularly as the few studies that have conducted multiple muscle tissue or multi-tissue biopsies suggest contradictory effects. For example, Leitner and Isely (1994) report an increase in mortalities, whereas other multiple tissue biopsy (Tyus *et al.*, 1999) and repeat biopsy studies (Hamilton *et al.*, 2002) report minimal interaction or repeat sampling effects. Further, given the paucity of assessments of long-term effects, trials focusing on growth and survival endpoints need to be completed. Of the biopsy studies summarised in Ackerson et al. (2014), less than half (42%) commented on survival over any time period and only 14% reported on growth-related effects. In the current study, the lack of measurable responses to a biopsy treatment over a fitness-relevant time scale (i.e. a feeding season) suggests that combining methods is feasible for post-spawned Atlantic salmon migrating to sea. This is promising considering that combining methods could enhance our overall understanding of Atlantic salmon marine ecology, including relationships between movement and feeding patterns and their consequences for fitness in terms of body condition and growth. The latter is particularly relevant given the established linkage between climate and ecosystem processes, which is believed to affect the abundance and productivity of Atlantic salmon populations (Mills *et al.*, 2013).
